# Multiscale RGB-Guided Fusion for Hyperspectral Image Super-Resolution

**DOI:** 10.3390/jimaging12020061

**Published:** 2026-01-28

**Authors:** Matteo Kolyszko, Marco Buzzelli, Simone Bianco, Raimondo Schettini

**Affiliations:** Department of Informatics, Systems and Communication, University of Milano-Bicocca, Viale Sarca 336, 20125 Milan, Italy; m.kolyszko@campus.unimib.it (M.K.); marco.buzzelli@unimib.it (M.B.);

**Keywords:** hyperspectral imaging, super-resolution, RGB guidance, deep learning, image fusion

## Abstract

Hyperspectral imaging (HSI) enables fine spectral analysis but is often limited by low spatial resolution due to sensor constraints. To address this, we propose CGNet, a color-guided hyperspectral super-resolution network that leverages complementary information from low-resolution hyperspectral inputs and high-resolution RGB images. CGNet adopts a dual-encoder design: the RGB encoder extracts hierarchical spatial features, while the HSI encoder progressively upsamples spectral features. A multi-scale fusion decoder then combines both modalities in a coarse-to-fine manner to reconstruct the high-resolution HSI. Training is driven by a hybrid loss that balances L1 and Spectral Angle Mapper (SAM), which ablation studies confirm as the most effective formulation. Experiments on two benchmarks, ARAD1K and StereoMSI, at ×4 and ×6 upscaling factors demonstrate that CGNet consistently outperforms state-of-the-art baselines. CGNet achieves higher PSNR and SSIM, lower SAM, and reduced $\Delta E_{00}$, confirming its ability to recover sharp spatial structures while preserving spectral fidelity.

## 1. Introduction

Hyperspectral imaging (HSI) captures dense spectral information across contiguous bands, enabling fine-grained material analysis with applications in remote sensing [1], precision agriculture [2], environmental monitoring [3], medical imaging [4], and cultural heritage preservation [5]. Compared to conventional RGB imaging, HSI offers unique advantages in terms of material discrimination and quantitative spectral analysis. However, the high spectral resolution of hyperspectral cameras typically comes at the expense of limited spatial resolution due to sensor and acquisition constraints. This trade-off reduces the effectiveness of hyperspectral data in tasks requiring both spatial detail and spectral fidelity.

Hyperspectral image super-resolution (HSI-SR) aims to reconstruct a high-resolution hyperspectral image (HR-HSI) from its low-resolution counterpart (LR-HSI). Traditional interpolation-based techniques, such as Bicubic interpolation, provide only marginal improvements, while deep learning-based single-image SR models, including RCAN [6] and EDSR [7], achieve better spatial quality but are not specifically tailored to preserve spectral properties, often leading to distortions as shown in [8]. Hyperspectral-specific networks such as SSPSR [8] improve spectral fidelity, but still struggle to maintain sharp spatial structures at higher upscaling factors as evidenced in [9].

A promising direction is to incorporate additional modalities to guide hyperspectral reconstruction. High-resolution RGB images, often available alongside hyperspectral measurements, provide complementary spatial and textural details that LR-HSI lacks. Several RGB-guided fusion approaches, including u2MDN [10], NonReg [11], and Integrated [12], have shown significant improvements by leveraging RGB guidance. Nonetheless, designing an architecture that effectively exploits spatial cues from RGB while simultaneously preserving spectral fidelity from HSI remains a challenging problem.

In this paper, we introduce CGNet, a color-guided hyperspectral super-resolution framework designed for natural-scene imagery, that integrates low-resolution hyperspectral data with high-resolution RGB observations. The architecture is based on a dual-encoder design and a multi-scale fusion decoder, enabling effective cross-modal feature interaction while preserving spectral fidelity and recovering fine spatial structures.

We summarize the main contributions of this work as follows:

- We develop a lightweight and efficient fusion mechanism that enables stable cross-modal integration across multiple spatial scales, improving spectral preservation without sacrificing spatial detail.
- We introduce a training strategy that balances pixel-wise and spectral objectives, L1 and Spectral Angle Mapper (SAM), and demonstrate through ablation that this balance substantially improves both spatial reconstruction and spectral accuracy.
- We conduct extensive experiments across multiple datasets, sensor types, and both reflectance and radiance-based domains, showing that CGNet maintains consistent performance and strong generalization in diverse real-world settings.

Overall, CGNet establishes a new state-of-the-art in hyperspectral super-resolution, achieving superior spatial detail recovery and spectral fidelity.

The rest of this paper is organized as follows: Section 2 reviews related works in hyperspectral super-resolution. Section 3 presents the architecture of CGNet and its building blocks. Section 4 describes the datasets, evaluation metrics, and implementation details. Quantitative and qualitative results are discussed in Section 5, followed by conclusions in Section 6.

## 2. Related Works

In this section, we review the most relevant studies on single HSI super-resolution and fusion-based HSI super-resolution.

### 2.1. Single HSI Super-Resolution

Single-image hyperspectral super-resolution is a long-standing and challenging problem, mainly because a single low-resolution (LR) hyperspectral observation can correspond to multiple plausible high-resolution (HR) solutions. This inherent ill-posedness has motivated the adoption of deep learning techniques, which have recently achieved remarkable progress in low-level vision tasks such as [13,14,15]. Specifically, convolutional neural networks (CNNs) have been widely employed to reconstruct HR hyperspectral images from their LR counterparts [8,13,16,17,18].

Early work by Mei et al. [13] proposed a three-dimensional fully convolutional architecture capable of exploiting both spatial and spectral correlations. Sun et al. [16] further introduced a feature pyramid strategy to capture multi-scale information. Jiang et al. [8] designed a method based on group convolution to better encode spatial-spectral priors, while Li et al. [17] explored hybrid 2D and 3D convolutional layers. In addition, Fu et al. [18] proposed a bidirectional 3D quasi-recurrent neural network (BiQRNN) to strengthen spectral dependency modeling.

More recently, transformer-based models [19,20,21] have opened new research directions: Hu et al. [22] and Liu et al. [23] explored their potential for hyperspectral restoration. Zhang et al. [9] introduced ESSAformer, which leverages spectral correlation coefficients and an efficient self-attention mechanism to enlarge the receptive field without significantly increasing computational cost. More recently, Zhang et al. [24] proposed a multiscale spatial–spectral CNN–Transformer network that integrates dilated convolutions for local multiscale feature extraction with a sparse spectral transformer to model global spectral dependencies in a coarse-to-fine manner. Nevertheless, the limited availability of hyperspectral training data and the lack of high-frequency spatial details in LR-HSI remain critical obstacles for single-image super-resolution methods.

### 2.2. Fusion-Based HSI Super-Resolution

Single-image hyperspectral super-resolution methods are fundamentally constrained by the absence of high-frequency spatial details in the low-resolution input. To alleviate this limitation, fusion-based techniques introduce an auxiliary modality, typically a high-resolution RGB or multispectral image, under the assumption that the additional data contain complementary spatial textures useful for enhancing the hyperspectral target. In most works, these methods require strictly aligned LR-HSI and HR-RGB (or MSI) pairs, which are generally synthesized by spatially and spectrally downsampling a high-resolution HSI.

Early research in this direction relied on prior-driven optimization. For example, sparse representation and dictionary learning methods were among the first attempts to inject high-resolution cues from RGB into HSI [25,26,27]. Matrix factorization [28] and tensor decomposition strategies [29] were also explored to jointly model spatial and spectral structures. Later studies incorporated manifold learning and Bayesian inference to better capture spatial–spectral dependencies [30,31].

With the advent of deep learning, CNN-based frameworks have become the mainstream solution. Several works designed end-to-end fusion pipelines to transfer spatial details from RGB into HSI while preserving spectral fidelity [32,33,34]. Attention mechanisms and degradation-aware modules have further improved robustness and reconstruction accuracy. For instance, Hu et al. [35] proposed a deep spatio–spectral attention CNN, while Guo et al. [36] introduced DAEM, which adaptively estimates degradation kernels during fusion. More recently, diffusion-based approaches such as HSR-Diff [37] refined HR-HSI outputs through iterative generative modeling. Transformer-based architectures have also been incorporated to better capture long-range dependencies between modalities [12,38,39]. More recently, Zhang et al. [40] proposed a dual-branch network with mutual guidance, where hyperspectral and multispectral features are processed in parallel and calibrated through bidirectional transformer-based attention. By explicitly reconciling modality differences before fusion, their approach improves spatial–spectral consistency while maintaining a relatively low computational cost. Recent advances in medical image analysis have demonstrated the effectiveness of multiscale and hierarchical feature fusion strategies for dense prediction and reconstruction tasks, particularly through scale-aware pyramidal representations that improve spatial coherence across resolution levels [41,42]. However, most medical imaging frameworks are developed for purely spatial or spatiotemporal data and are optimized for tasks such as segmentation, classification, or structural reconstruction, without explicitly addressing spectral consistency. In hyperspectral super-resolution, preserving the geometric relationships between spectral bands under spatial upsampling is a fundamental requirement that goes beyond spatial accuracy alone. While the proposed framework shares a high-level multiscale design philosophy with recent medical imaging approaches, it differs substantially in its fusion strategy and supervision, which are explicitly designed to protect spectral fidelity through scale-aligned cross-modal interactions and spectral-angle-based constraints. As such, the proposed method should be regarded as complementary to, rather than a direct extension of, existing multiscale medical imaging models.

The proposed CGNet falls within the class of fusion-based RGB-guided hyperspectral super-resolution methods, which exploit the complementary characteristics of RGB and hyperspectral data to recover high-resolution images with both rich spatial detail and accurate spectral information. At a high level, CGNet shares common design principles with existing RGB-guided architectures, such as the use of parallel processing streams for RGB and hyperspectral inputs. However, it departs from prior approaches in the way fusion, scale alignment, and spectral preservation are explicitly handled.

While several existing methods adopt dual-stream designs to separately extract spatial and spectral features, CGNet introduces a more structured multiscale interaction between modalities. This design enables effective information exchange across resolutions while maintaining strict control over spectral fidelity.

Overall, CGNet differs from previous RGB-guided approaches in three main aspects:

- Fusion strategy: explicit multiscale, coarse-to-fine feature fusion.
- Scale alignment: hierarchical resolution matching via progressive upsampling.
- Spectral protection: dedicated hyperspectral encoding combined with SAM-based supervision.

## 3. Proposed Method

**Figure 1:**
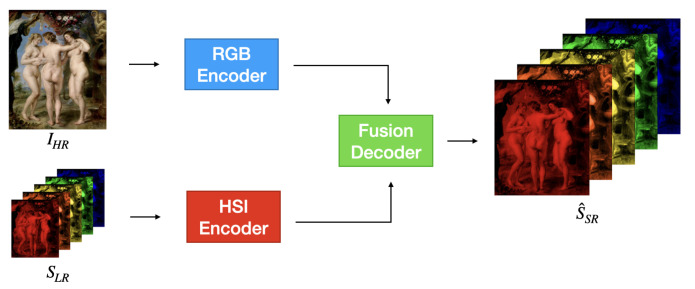
Overview of the proposed CGNet architecture. The network takes as input a low-resolution hyperspectral image and a high-resolution RGB image. It employs two parallel encoders to extract multiscale spectral and spatial features, which are progressively fused in a coarse-to-fine manner by the fusion decoder. The final output is a super-resolved hyperspectral image with full spatial and spectral fidelity.

In this section, we first formulate the problem of RGB-guided hyperspectral super-resolution. We then present the overall architecture of CGNet and describe its three main components: the RGB encoder, the HSI encoder, and the fusion decoder. An overview of the framework is shown in Figure 1.

### 3.1. Problem Formulation

Let $S_{\mathrm{LR}}\in R^{C\times h\times w}$ be a low-resolution hyperspectral image with *C* spectral bands and spatial dimensions (h,w), and let $I_{\mathrm{HR}}\in R^{3\times H\times W}$ be a high-resolution RGB image of the same scene, where the following applies:

(H,W)=(r·h,r·w),r∈{4,6}.

The goal is to recover the corresponding high-resolution hyperspectral cube $S_{\mathrm{HR}}\in R^{C\times H\times W}$, preserving the spectral information of $S_{\mathrm{LR}}$ while restoring the spatial details available in $I_{\mathrm{HR}}$.

We formulate this task as learning a parametric function $F_{\theta }\left(\cdot \right)$ such that the following applies:

(1) $${\hat{S}}_{\mathrm{SR}}=F_{\theta }\left(S_{\mathrm{LR}},\;I_{\mathrm{HR}}\right)\;\approx \;S_{\mathrm{HR}},$$

where ${\hat{S}}_{\mathrm{SR}}$ denotes the reconstructed high-resolution hyperspectral image and θ are the learnable parameters. The model parameters are optimized using a loss function that balances spatial and spectral accuracy, as detailed in Section 5.4.1.

### 3.2. Model Architecture

CGNet is a color-guided hyperspectral super-resolution architecture that fuses low-resolution hyperspectral data $S_{\mathrm{LR}}$ with high-resolution RGB guidance $I_{\mathrm{HR}}$ to produce a high-resolution hyperspectral estimate ${\hat{S}}_{\mathrm{SR}}$. As illustrated in Figure 1, the network processes the two inputs with parallel encoders and fuses their representations through a multiscale decoder.

CGNet is composed of three main components:

- RGB Encoder: Extracts a hierarchy of spatial features from $I_{\mathrm{HR}}$, capturing fine textures and structural cues at multiple resolutions.
- HSI Encoder: Extracts spectrally rich features from $S_{\mathrm{LR}}$ and progressively upsamples them to match the spatial resolutions of the RGB features.
- Fusion Decoder: Integrates multiscale features from both encoders in a coarse-to-fine manner and reconstructs the final high-resolution hyperspectral image.

The overall forward pass can be written as follows:

(2) $${\hat{S}}_{\mathrm{SR}}=D(E_{\mathrm{HSI}}\left(S_{\mathrm{LR}}\right),\;E_{\mathrm{RGB}}\left(I_{\mathrm{HR}}\right)),$$

where $E_{\mathrm{HSI}}\left(\cdot \right)$ and $E_{\mathrm{RGB}}\left(\cdot \right)$, respectively, denote the HSI and RGB encoders, and D(·,·) denotes the fusion decoder.

**Figure 2:**
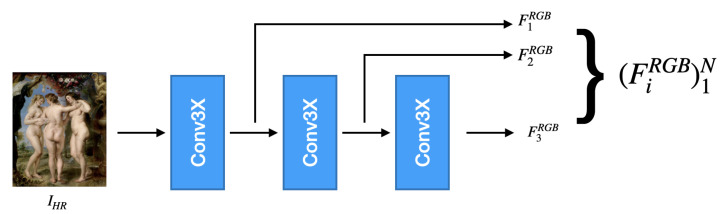
Architecture of the proposed RGB encoder $\left(E_{\mathrm{RGB}}\right)$. The module takes as input the high-resolution RGB image and extracts spatial features at multiple scales through a sequence of convolutional blocks with increasing stride. These hierarchical features capture both fine-grained details and broader contextual information, and are used to guide the reconstruction process in the decoder.

**RGB Encoder.** The RGB encoder $E_{\mathrm{RGB}}$, illustrated in Figure 2, extracts a hierarchy of spatial features from the high-resolution RGB input $I_{\mathrm{HR}}\in R^{3\times H\times W}$. Its role is to capture fine textures, edges, and structural patterns at multiple scales, which serve as spatial guidance during hyperspectral reconstruction.

Each stage of the encoder is implemented using the Conv3XC block introduced by Wan et al. [43]. Let C(·;ρ) denote such a block with stride ρ. The encoder applies three sequential transformations:

(3) $$\begin{array}{cc} F_{1}^{\mathrm{RGB}} & =C(I_{\mathrm{HR}};\;\rho =1), \end{array}$$

(4) $$\begin{array}{cc} F_{2}^{\mathrm{RGB}} & =C(F_{1}^{\mathrm{RGB}};\;\rho =2), \end{array}$$

(5) $$\begin{array}{cc} F_{3}^{\mathrm{RGB}} & =C(F_{2}^{\mathrm{RGB}};\;\rho =2), \end{array}$$

which produce the multiscale feature pyramid:

(6) $$E_{\mathrm{RGB}}\left(I_{\mathrm{HR}}\right)=\left\{F_{1}^{\mathrm{RGB}},\;F_{2}^{\mathrm{RGB}},\;F_{3}^{\mathrm{RGB}}\right\}.$$

The feature maps have the following spatial resolutions:

(7) $$\begin{array}{cc} F_{1}^{\mathrm{RGB}} & \in R^{C\times H\times W}, \end{array}$$

(8) $$\begin{array}{cc} F_{2}^{\mathrm{RGB}} & \in R^{C\times \frac{H}{2}\times \frac{W}{2}}, \end{array}$$

(9) $$\begin{array}{cc} F_{3}^{\mathrm{RGB}} & \in R^{C\times \frac{H}{4}\times \frac{W}{4}}, \end{array}$$

where all feature maps share the same number of channels C=64. The first stage preserves full spatial detail and captures fine-grained RGB textures. The subsequent downsampling stages increase the receptive field and extract progressively higher-level structures.

**Figure 3:**
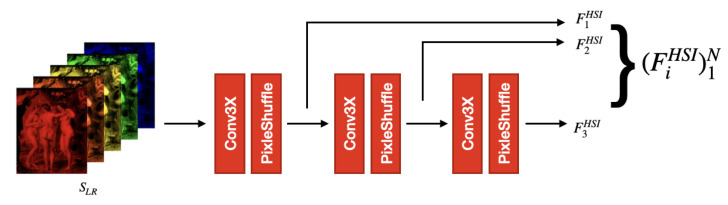
Architecture of the proposed HSI encoder $\left(E_{\mathrm{HSI}}\right)$. The encoder extracts low-level spectral features from the input hyperspectral image and progressively upsamples them using convolution and PixelShuffle operations. The resulting multiscale representations match the spatial resolutions of the RGB features and enable effective cross-modal fusion in the decoder.

**HSI Encoder.** The HSI encoder $E_{\mathrm{HSI}}$, shown in Figure 3, is designed to extract spectrally informative features from the low-resolution hyperspectral input $S_{\mathrm{LR}}$ and to progressively upsample them to higher spatial resolutions. This module complements the RGB encoder by providing spectral context at multiple scales that can be aligned with the RGB feature maps in the decoder.

Let C(·;ρ) denote a Conv3XC block with stride ρ, and let U(·) denote an upsampling block composed of a 5×5 convolution followed by PixelShuffle [44]. The HSI encoder applies the following sequence of operations:

(10) $$\begin{array}{cc} F_{1}^{\mathrm{HSI}} & =C(S_{\mathrm{LR}};\;\rho =1), \end{array}$$

(11) $$\begin{array}{cc} F_{2}^{\mathrm{HSI}} & =U(F_{1}^{\mathrm{HSI}}), \end{array}$$

(12) $$\begin{array}{cc} F_{3}^{\mathrm{HSI}} & =U(F_{2}^{\mathrm{HSI}}), \end{array}$$

and outputs the multiscale feature pyramid

(13) $$E_{\mathrm{HSI}}\left(S_{\mathrm{LR}}\right)=\left\{F_{1}^{\mathrm{HSI}},\;F_{2}^{\mathrm{HSI}},\;F_{3}^{\mathrm{HSI}}\right\}.$$

The three feature maps have the following spatial resolutions:

(14) $$\begin{array}{cc} F_{1}^{\mathrm{HSI}} & \in R^{C\times h\times w}, \end{array}$$

(15) $$\begin{array}{cc} F_{2}^{\mathrm{HSI}} & \in R^{C\times 2h\times 2w}, \end{array}$$

(16) $$\begin{array}{cc} F_{3}^{\mathrm{HSI}} & \in R^{C\times 4h\times 4w}, \end{array}$$

where all feature maps share the same number of channels C=64. This design simplifies cross-modal fusion with the RGB feature maps $F_{3}^{\mathrm{RGB}}$, $F_{2}^{\mathrm{RGB}}$, and $F_{1}^{\mathrm{RGB}}$, which are defined at matching spatial resolutions.

Overall, the hierarchical upsampling structure of $E_{\mathrm{HSI}}$ enables the network to propagate rich spectral information from the original low-resolution grid to increasingly finer spatial domains. In this way, the hyperspectral features can be effectively aligned with the RGB guidance at each scale, providing a strong spectral prior for the fusion decoder.

**Figure 4:**
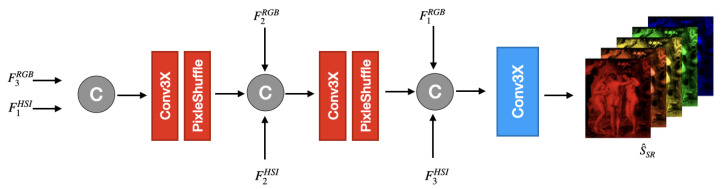
Overview of the fusion decoder module. Given multiscale feature pyramids from the HSI and RGB encoders, the decoder performs a progressive fusion and upsampling across resolution levels. At each stage, features are concatenated and processed through a PixelShuffle-based upsampling block. The final output is generated by a convolutional reconstruction block that produces the high-resolution hyperspectral image.

**Fusion Decoder.** The fusion decoder D(·), illustrated in Figure 4, reconstructs the high-resolution hyperspectral output by progressively merging the multiscale feature representations extracted by the two encoders. Let $\left\{F_{1}^{\mathrm{HSI}},F_{2}^{\mathrm{HSI}},F_{3}^{\mathrm{HSI}}\right\}$ denote the features from the HSI encoder and $\left\{F_{1}^{\mathrm{RGB}},F_{2}^{\mathrm{RGB}},F_{3}^{\mathrm{RGB}}\right\}$ those from the RGB encoder.

The decoding process is formulated as follows:

(17) $${\hat{S}}_{\mathrm{SR}}=D\;\left({\left\{F_{i}^{\mathrm{HSI}}\right\}}_{i=1}^{3},\;{\left\{F_{j}^{\mathrm{RGB}}\right\}}_{j=3}^{1}\right),$$

where ${\hat{S}}_{\mathrm{SR}}\in R^{C\times H\times W}$ denotes the reconstructed high-resolution hyperspectral cube.

The lowest-resolution hyperspectral feature $F_{1}^{\mathrm{HSI}}\in R^{C\times h\times w}$ is concatenated with the coarsest RGB feature $F_{3}^{\mathrm{RGB}}\in R^{C\times h\times w}$. The fused tensor is processed by an upsampling block U(·), composed of a Conv3XC module followed by PixelShuffle:

(18) $$x_{1}=U\;\left(\mathrm{Concat}\;\left(F_{1}^{\mathrm{HSI}},\;F_{3}^{\mathrm{RGB}}\right)\right).$$

The upsampled feature $x_{1}$ is aligned with $F_{2}^{\mathrm{HSI}}$ and $F_{2}^{\mathrm{RGB}}$. The three tensors are concatenated and processed through a second upsampling block:

(19) $$x_{2}=U\;\left(\mathrm{Concat}\;\left(x_{1},\;F_{2}^{\mathrm{HSI}},\;F_{2}^{\mathrm{RGB}}\right)\right).$$

At the highest resolution, $x_{2}$ is concatenated with the finest hyperspectral feature $F_{3}^{\mathrm{HSI}}$ and the full-resolution RGB feature $F_{1}^{\mathrm{RGB}}$. A final Conv3XC block with stride p=1 produces the reconstructed output:

(20) $${\hat{S}}_{\mathrm{SR}}=C\;\left(\mathrm{Concat}\;\left(x_{2},\;F_{3}^{\mathrm{HSI}},\;F_{1}^{\mathrm{RGB}}\right);\;p=1\right).$$

This yields the final super-resolved hyperspectral estimate ${\hat{S}}_{\mathrm{SR}}\in R^{C\times H\times W}$.

The fusion strategy adopted in the decoder is motivated by the different semantic properties of features extracted at different depths in the RGB and hyperspectral encoders. Shallow features primarily encode low-level spatial details, such as edges and textures, while deeper features capture more abstract and context-aware representations. In the RGB branch, deeper features provide robust structural cues with reduced sensitivity to local noise, whereas shallow RGB features preserve fine spatial details. Conversely, in the hyperspectral branch, shallow features retain detailed spectral information, while deeper features encode more global spectral–spatial context. Based on this observation, the decoder pairs deep RGB features with shallow hyperspectral features at coarse resolutions to inject reliable structural guidance while preserving fine-grained spectral information. At higher resolutions, shallow RGB features are paired with deeper hyperspectral features to refine spatial details while maintaining globally consistent spectral representations. The fusion operation itself is implemented via simple feature concatenation; therefore, the effectiveness of the decoder arises from the cross-level feature pairing rather than from a complex fusion operator. This cross-level interaction exploits the complementary strengths of RGB and hyperspectral modalities at different semantic levels, enabling effective spatial enhancement without compromising spectral fidelity.

Regarding architectural hyperparameters, the design choices are guided by a trade-off between reconstruction accuracy and computational efficiency. The number of feature channels is kept moderate and fixed to 64 across the network, as the proposed multiscale fusion strategy and Conv3XC blocks provide sufficient representational capacity without requiring excessively wide feature maps. Kernel sizes are set to 3×3, which allows preserving local spatial structures while maintaining computational efficiency. Instead of relying on larger kernels to increase the receptive field, CGNet enlarges the effective receptive field through hierarchical multiscale processing and progressive fusion across resolutions. Finally, the number of scales is determined by the target upsampling factor and by the need to perform resolution enhancement progressively rather than in a single step. In the current design, two upsampling stages are employed, each with a limited per-stage scaling factor, avoiding a direct large-scale increase (e.g., ×4) in one operation. This progressive strategy stabilizes training and enables more effective cross-modal feature alignment at intermediate resolutions.

## 4. Experimental Setup

In this section, we describe the experimental setup used to evaluate our method. We present the datasets, the evaluation metrics, and the implementation details.

### 4.1. Datasets

Our approach is evaluated on two benchmark hyperspectral datasets, which span a variety of acquisition conditions, spectral resolutions, and scene categories: ARAD1K and StereoMSI.

#### 4.1.1. ARAD1K Dataset

The ARAD1K dataset was introduced as part of the NTIRE 2022 Spectral Recovery Challenge [45] and remains one of the most comprehensive datasets for spectral image reconstruction. It includes 1000 real-world hyperspectral scenes, captured using a Specim IQ push-broom camera. Each scene features a native resolution of 512×512 pixels and originally comprises 204 spectral channels ranging from 400 nm to 1000 nm. For the purpose of spectral reconstruction in the visible range, the data is preprocessed through radiometric calibration and uniformly downsampled to 31 bands in the 400–700 nm range (with a 10 nm step), resulting in final image dimensions of 482×512×31. Since the original test split does not include corresponding hyperspectral labels, we adopt a modified partitioning of the dataset, using 850 samples for training, 50 for validation, and 50 for testing.

#### 4.1.2. StereoMSI Dataset

The StereoMSI dataset, released as part of the PIRM 2018 Spectral Image Super-Resolution Challenge [46], serves as a standard benchmark for evaluating both RGB-guided and single-sensor spectral super-resolution techniques. It comprises 350 stereo pairs consisting of RGB and multispectral images, recorded across a diverse set of natural and man-made environments such as forests, offices, industrial zones, and desert areas near Canberra, Australia. Each stereo pair consists of a high-resolution RGB image with a resolution of 960×480 pixels, and a corresponding low-resolution multispectral image of 480×240 pixels. The spectral image is acquired using a snapshot sensor based on a 4×4 mosaic filter array, yielding 16 effective spectral bands within the visible spectrum (470–620 nm).

### 4.2. Evaluation Metrics

To assess the effectiveness of the proposed spectral super-resolution method, we adopt three widely recognized evaluation metrics [47]: Spectral Angle Mapper (SAM) [48], Peak Signal-to-Noise Ratio (PSNR), and Structural Similarity Index Measure (SSIM) [49]. These metrics capture complementary aspects of reconstruction quality, ranging from spectral accuracy (SAM) to per-band reconstruction fidelity (PSNR) and structural similarity across spectral channels (SSIM), thus providing a comprehensive evaluation framework. Moreover, the same metrics are consistently adopted by recent state-of-the-art RGB-guided hyperspectral super-resolution methods evaluated on ARAD1K and StereoMSI, enabling a fair and direct comparison under a unified experimental protocol [10,11,12].

#### 4.2.1. Spectral Angle Mapper (SAM)

The Spectral Angle Mapper (SAM) is a widely adopted metric in hyperspectral image analysis for assessing spectral consistency between a reconstructed pixel and its ground-truth counterpart. It quantifies the angle between two spectral vectors, treating them as points in a high-dimensional space. As such, it focuses more on the shape of the spectra than on their absolute values. Given a ground-truth spectrum r and a predicted spectrum $\hat{r}$, SAM is defined as follows:

(21) $$\mathrm{SAM}\left(r,\hat{r}\right)=\cos^{-1}\left(\frac{\langle r,\hat{r}\rangle }{{\left|r\right|}_{2}\cdot {\left|\hat{r}\right|}_{2}}\right).$$

The SAM is measured in radians. The computation is applied pixel-wise, and the average SAM over all valid pixels in an image is taken as the image-level score. The final reported value corresponds to the mean SAM across the test set. Lower values indicate better spectral alignment between prediction and ground truth.

#### 4.2.2. Peak Signal-to-Noise Ratio (PSNR)

PSNR quantifies the pixel-level reconstruction accuracy by evaluating the ratio between the peak possible signal value and the magnitude of reconstruction errors. It is derived from the Mean Squared Error (MSE) between the predicted hyperspectral image $\hat{I}\in R^{H\times W\times C}$ and the corresponding ground truth $I\in R^{H\times W\times C}$, where *H*, *W*, and *C* represent the spatial height, width, and number of spectral bands, respectively.

The MSE is calculated as follows:

(22) $$\mathrm{MSE}=\frac{1}{HWC}\sum_{i=1}^{H}\sum_{j=1}^{W}\sum_{c=1}^{C}{\left({\hat{I}}_{i,j,c}-I_{i,j,c}\right)}^{2}$$

From the MSE, the PSNR is computed in decibels (dB) as follows:

(23) $$\mathrm{PSNR}=10\cdot \log_{10}\left(\frac{MAX^{2}}{\mathrm{MSE}}\right)$$

where MAX denotes the maximum possible pixel value (typically 1.0 for normalized reflectance or radiance data). A higher PSNR indicates smaller reconstruction errors and thus better fidelity to the ground truth.

#### 4.2.3. Structural Similarity Index (SSIM)

The Structural Similarity Index Measure (SSIM) is a perceptual metric that evaluates the similarity between two images based on luminance, contrast, and structural information. Although originally introduced for grayscale images, SSIM is widely adopted for assessing multi-channel data such as hyperspectral or multispectral images.

In our case, SSIM is computed independently on each spectral band and then averaged to produce a single scalar score. Given a ground-truth patch *I* and a reconstructed patch $\hat{I}$, SSIM is formulated as follows:

(24) $$\mathrm{SSIM}\left(I,\hat{I}\right)=\frac{\left(2\mu_{I}\mu_{\hat{I}}+C_{1}\right)\left(2\sigma_{I\hat{I}}+C_{2}\right)}{\left(\mu_{I}^{2}+\mu_{\hat{I}}^{2}+C_{1}\right)\left(\sigma_{I}^{2}+\sigma_{\hat{I}}^{2}+C_{2}\right)},$$

where $\mu_{I}$ and $\mu_{\hat{I}}$ are the local means, $\sigma_{I}^{2}$ and $\sigma_{\hat{I}}^{2}$ are the local variances, and $\sigma_{I\hat{I}}$ is the local covariance between the reference and predicted patches. The constants $C_{1}$ and $C_{2}$ are small regularization terms to ensure numerical stability.

SSIM is calculated using a sliding Gaussian window (typically of size 11×11) over each spectral channel. The final SSIM score is obtained by averaging spatially across all pixels and spectrally across all channels. The metric ranges from 0 to 1, where values closer to 1 indicate higher structural similarity between the predicted and reference images.

#### 4.2.4. Color Difference Metric ($\Delta E_{00}$)

To assess the perceptual color fidelity between the reconstructed and reference hyperspectral images, we compute the DeltaE2000 ($\Delta E_{00}$) color difference metric, which is widely used in color science for quantifying human-visible discrepancies.

The $\Delta E_{00}$ value measures the perceptual distance between two colors represented in the CIE Lab color space. Given two spectra—one from the ground truth and one reconstructed—we first convert them to tristimulus values using a standard observer model (e.g., CIE 1931 2°) and an illuminant (typically D65), and then transform them to the CIE Lab space. The perceptual color difference is then computed according to the CIEDE2000 formula, which accounts for improvements over previous models by including corrections for lightness, chroma, and hue.

For two colors with Lab coordinates $\left(L_{1},a_{1},b_{1}\right)$ and $\left(L_{2},a_{2},b_{2}\right)$, the $\Delta E_{00}$ is computed as follows:

(25) $$\Delta E_{00}=\sqrt{{\left(\frac{\Delta L^{\prime }}{k_{L}S_{L}}\right)}^{2}+{\left(\frac{\Delta C^{\prime }}{k_{C}S_{C}}\right)}^{2}+{\left(\frac{\Delta H^{\prime }}{k_{H}S_{H}}\right)}^{2}+R_{T}\left(\frac{\Delta C^{\prime }}{k_{C}S_{C}}\right)\left(\frac{\Delta H^{\prime }}{k_{H}S_{H}}\right)}$$

where $\Delta L^{\prime }$, $\Delta C^{\prime }$, and $\Delta H^{\prime }$ represent the differences in lightness, chroma, and hue, respectively. The terms $S_{L}$, $S_{C}$, and $S_{H}$ are weighting functions, $k_{L}=k_{C}=k_{H}=1$ are parametric factors (in most settings), and $R_{T}$ is a rotation term that accounts for the interaction between chroma and hue.

The $\Delta E_{00}$ score is computed per pixel after spectral-to-RGB conversion and averaged across the image. Lower values of $\Delta E_{00}$ indicate better colorimetric agreement, with values below 1 generally considered imperceptible to the human eye.

### 4.3. Implementation Details

The proposed CGNet network was implemented in PyTorch version 2.10 and trained on a single NVIDIA TITAN X GPU. Training was carried out for a maximum of 1000 epochs; however, an early stopping criterion was employed to prevent overfitting, terminating the optimization process when the validation loss failed to improve for 100 consecutive epochs. With this strategy, training converged after 560 epochs, requiring approximately 21 h in total.

Model parameters were optimized using the Adam algorithm, with an initial learning rate of $5\times 10^{-4}$ and a weight decay of $1\times 10^{-5}$. The learning rate was dynamically reduced by a factor of 0.5 whenever the validation loss did not decrease for 50 successive epochs.

As training objective, we employed a combined loss function defined as follows:

(26) $$L=\alpha \cdot L_{L1}+\left(1-\alpha \right)\cdot L_{\mathrm{SAM}},$$

with α=0.7. This configuration was selected based on the results of our ablation study presented in Section 5.4.1, which demonstrated that it provides the best trade-off between spatial reconstruction accuracy and spectral fidelity.

## 5. Results

We evaluate CGNet on two hyperspectral benchmarks, ARAD1K and StereoMSI, reporting results at upscaling factors ×4 and ×6. The comparison includes classical interpolation (Bicubic), single-image super-resolution networks originally designed for RGB images (RCAN [6], EDSR [7]), a hyperspectral-specific method (SSPSR [8]), and RGB-guided fusion approaches (u2MDN [10], NonReg [11], Integrated [12]), alongside our model. To ensure a fair comparison, each baseline method is trained and evaluated under two configurations: the original training setting reported by the respective authors and a unified training pipeline identical to that of CGNet. In the unified training setting, the same optimization strategy, learning rate schedule, early stopping criterion, and learning rate decay strategy are applied to all methods, including CGNet and the baseline models, with identical monitoring metrics and patience values. For each method, we report the best performance achieved across the two configurations. Quantitative comparisons on ARAD1K are summarized in Table 1, while results on StereoMSI are reported in Table 2; metrics include SAM, PSNR, SSIM, and $\Delta E_{00}$.

### 5.1. Results on ARAD1K and StereoMSI

Table 1 and Table 2 summarize the quantitative performance on ARAD1K and StereoMSI, respectively. Across both datasets, single-image baselines (Bicubic, RCAN [6], SSPSR [8], EDSR [7]) achieve only moderate reconstruction quality. Relying solely on LR-HSI, these models fail to recover high-frequency spatial structures and often introduce spectral distortions, leading to lower PSNR/SSIM and substantially higher SAM and $\Delta E_{00}$ scores. This confirms the ill-posedness of hyperspectral super-resolution without auxiliary guidance.

Methods that incorporate RGB information exhibit clear and consistent gains across both datasets. Fusion-based approaches such as u2MDN [10], NonReg [11], and Integrated [12] leverage the high-resolution spatial priors provided by the RGB modality, resulting in improved detail reconstruction and enhanced spectral consistency. These improvements are reflected in reduced SAM and $\Delta E_{00}$ values. Our model achieves the best performance on both ARAD1K and StereoMSI at both the upscaling factors ×4 and ×6. Relative to the strongest competing methods, it consistently delivers higher PSNR/SSIM and lower SAM and $\Delta E_{00}$. The advantage is particularly pronounced at ×6, where competing approaches exhibit a sharper decline in reconstruction fidelity.

**Figure 5:**
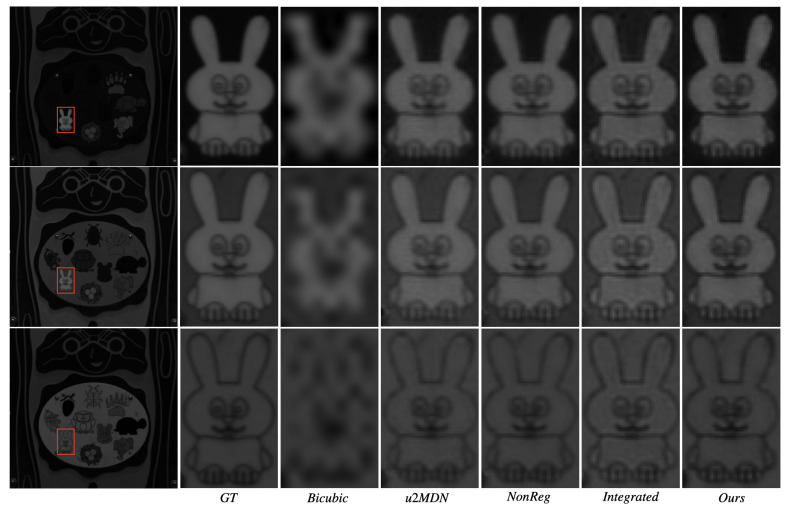
Visual comparison of reconstructed hyperspectral images from ARAD1K at wavelengths 480 nm, 580 nm, and 680 nm, under ×6 super-resolution. Rows correspond to spectral bands; columns to different methods.

**Figure 6:**
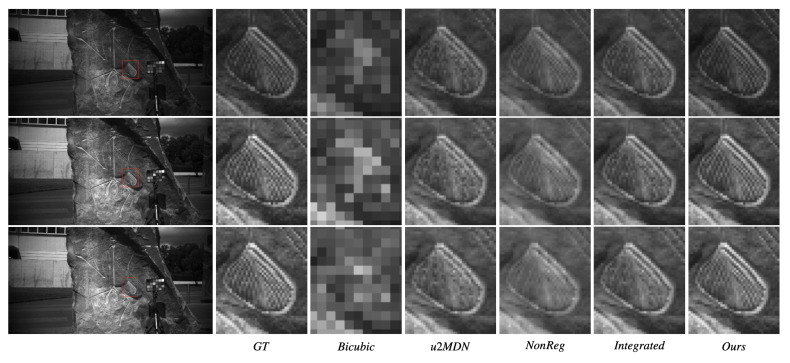
Visual comparison of reconstructed hyperspectral images from StereoMSI at wavelengths 477 nm, 537 nm, and 617 nm, under ×6 super-resolution. Rows correspond to spectral bands; columns to different methods.

**Figure 7:**
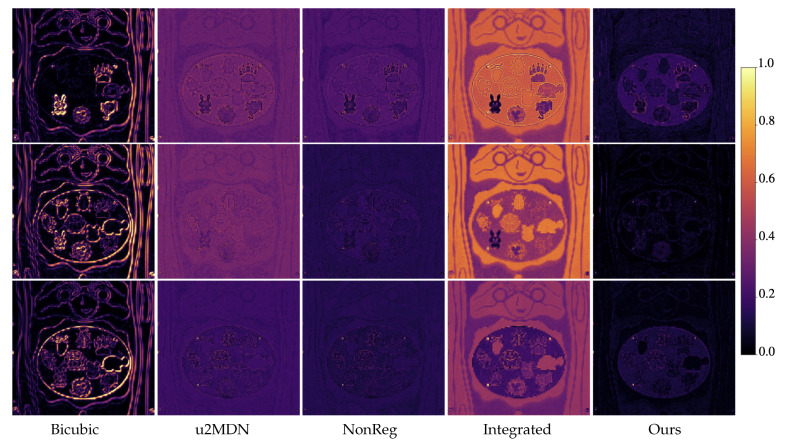
Error maps from ARAD1K at wavelengths 480 nm, 580 nm, and 680 nm, under ×6 super-resolution. Rows correspond to spectral bands; columns to different methods. Brighter regions represent higher reconstruction errors, highlighting differences in spatial accuracy among the models.

**Figure 8:**
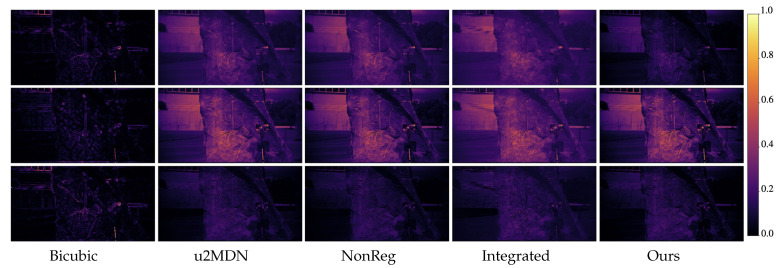
Error maps from StereoMSI at wavelengths 477 nm, 537 nm, and 617 nm, under ×6 super-resolution. Rows correspond to spectral bands; columns to different methods. Brighter regions represent higher reconstruction errors, highlighting differences in spatial accuracy among the models.

Figure 5 and Figure 6 visually compare spatial reconstructions. From the reported images, it is possible to observe that several competing methods tend to produce smoother textures or exhibit distortions in fine structural details, whereas our approach better preserves edges and small objects. Error maps (Figure 7 and Figure 8) further demonstrate that our method generates lower and more spatially uniform residuals, indicating improved cross-band stability.

**Figure 9:**
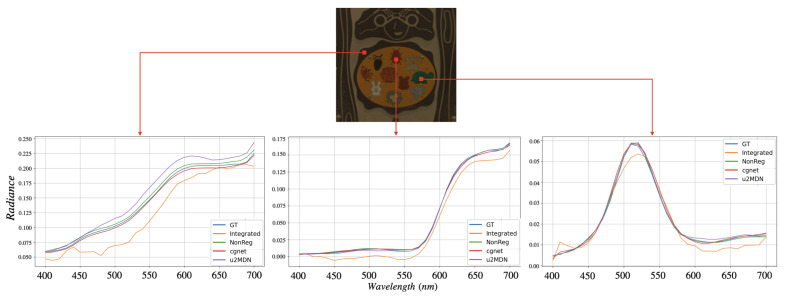
Spectral evaluation of reconstructed HSIs on ARAD1K at ×6 super-resolution. The **top** panel shows an sRGB rendering of the reconstructed image with three representative pixels marked. The **bottom** panel reports the spectral signatures corresponding to these pixels, comparing different methods with the ground truth.

**Figure 10:**
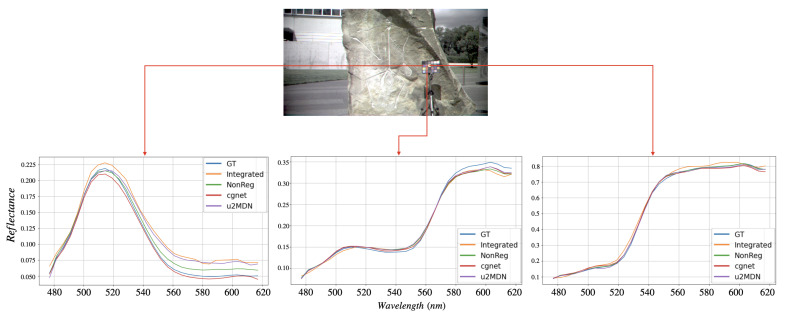
Spectral evaluation of reconstructed HSIs on StereoMSI at ×6 super-resolution. The **top** panel shows an sRGB rendering of the reconstructed image with three representative pixels marked. The **bottom** panel reports the spectral signatures corresponding to these pixels, comparing different methods with the ground truth.

Finally, pixel-wise spectral plots in Figure 9 and Figure 10 confirm that our model yields spectral signatures closely matching the ground truth across the entire visible range. While other models typically diverge beyond 550–600 nm, our method consistently provides more accurate reconstructions in this wavelength range. Overall, the results on ARAD1K and StereoMSI demonstrate that the proposed framework achieves state-of-the-art performance and generalizes effectively across datasets with different characteristics—recovering fine spatial detail while preserving high spectral fidelity.

### 5.2. Cross-Dataset Generalization

To evaluate the generalization capability of CGNet, we conduct a cross-dataset experiment where the model is trained on ARAD1K and directly tested on StereoMSI without any fine-tuning.

Table 3 reports the cross-dataset generalization results obtained by training the models on ARAD1K and directly testing them on StereoMSI without any fine-tuning. This experiment represents a challenging setting due to the significant domain shift between the two datasets, including differences in sensor characteristics, spectral sampling, and scene content. As expected, all methods experience a noticeable degradation in reconstruction quality under cross-dataset evaluation. In particular, PSNR decreases by approximately 8 dB for all approaches, indicating the difficulty of transferring spatial reconstruction capabilities across datasets. Nevertheless, CGNet consistently achieves the highest PSNR and SSIM at both ×4 and ×6 scales, suggesting superior preservation of spatial structures even in the presence of severe domain shift. Notably, CGNet exhibits a smaller drop in SSIM compared to competing methods, especially at ×4, where the degradation is limited to 7 points versus 8 points for other models, and at ×6, where the drop remains lower than that of competing approaches. This behavior indicates improved structural generalization, likely due to the explicit multiscale fusion strategy and the separation of spatial and spectral processing streams. Overall, while cross-dataset evaluation leads to an inevitable performance drop for all methods, CGNet consistently exhibits a smaller degradation and retains a clear advantage over competing RGB-guided approaches. These results confirm that the proposed architecture generalizes more effectively across datasets with different spectral and spatial characteristics, validating the robustness of the dual-encoder and multiscale fusion design.

### 5.3. Computational Efficiency Analysis

Table 4 presents a detailed comparison between reconstruction accuracy and computational efficiency at the ×4 scale on the ARAD1K dataset. While several color-guided methods achieve strong reconstruction performance, their practical applicability is often constrained by high inference time or excessive computational overhead.

Despite having a higher number of parameters and GFLOPs compared to some lightweight architectures, the proposed method achieves the second fastest inference time among all learning-based approaches. This result highlights that computational efficiency in practice is not solely determined by model size or theoretical complexity, but also by architectural design and data flow efficiency.

In particular, while NonReg attains the lowest runtime due to its highly optimized non-iterative formulation, our model substantially outperforms it in terms of spectral and spatial reconstruction accuracy, achieving the best overall SAM, PSNR, and SSIM. Compared to u2MDN, which exhibits extremely low parameter count and GFLOPs, our approach is significantly faster at inference, demonstrating superior hardware efficiency despite higher nominal complexity.

Overall, the proposed method achieves an advantageous balance between reconstruction quality and real-world inference efficiency, making it suitable for practical high-resolution hyperspectral imaging applications.

### 5.4. Ablation Study

Three ablation studies are conducted to thoroughly understand the proposed network. All the experiments are based on the Arad1K dataset with a scale factor of ×4.

#### 5.4.1. Ablation Study on Loss Function

We further investigate the impact of different loss formulations on the performance of the proposed framework. In particular, we compare:

1. A weighted combination of pixel-wise L1 loss and the Spectral Angle Mapper (SAM) loss:
  (27) $$L_{L1+\mathrm{SAM}}=\alpha \cdot L_{L1}+\left(1-\alpha \right)\cdot L_{\mathrm{SAM}},$$
  with different values of α∈{0.3,0.5,0.7};
2. Pixel-wise L1 loss alone, corresponding to the special case of the previous formulation with α=1:
  (28) $$L_{L1}={∥\hat{H}-H_{\mathrm{HR}}∥}_{1};$$
3. A regularization strategy based on spatial and spectral total variation (TV) losses:
  (29) $$L_{\mathrm{TV}}=L_{\mathrm{TV}}^{\mathrm{spatial}}+L_{\mathrm{TV}}^{\mathrm{spectral}},$$
  where given a hyperspectral image $X\in R^{H\times W\times K}$, the spatial TV term is defined as follows:
  (30) $$L_{\mathrm{TV}}^{\mathrm{spatial}}=\sum_{i,j,k}\left(\left|X_{i+1,j,k}-X_{i,j,k}\right|+\left|X_{i,j+1,k}-X_{i,j,k}\right|\right),$$
  and the spectral TV term as follows:
  (31) $$L_{\mathrm{TV}}^{\mathrm{spectral}}=\sum_{i,j,k}\left|X_{i,j,k+1}-X_{i,j,k}\right|.$$
  Spatial TV promotes piecewise-smooth structures while preserving edges across the spatial domain, whereas spectral TV enforces smooth evolution across adjacent wavelength channels, consistent with the natural behavior of reflectance spectra.

Beyond empirical evaluation, the complementarity between the L1 and SAM losses can be explained by their distinct behaviors at the gradient level. The SAM loss operates on the angular similarity between spectral vectors and is inherently scale-invariant, as it depends only on the normalized direction of the spectra. Consequently, its gradients primarily enforce consistency in spectral shape, encouraging the preservation of relative inter-band relationships regardless of absolute intensity. This property is particularly important for hyperspectral reconstruction, where accurate spectral signatures are more critical than exact pixel-wise magnitudes. In contrast, the L1 loss provides pixel-wise supervision with gradients directly proportional to local reconstruction errors. This leads to stable optimization and strongly penalizes absolute deviations, making L1 especially effective at preserving spatial structures and sharp edges in the reconstructed images. However, when used alone, L1 does not explicitly constrain the spectral geometry, which can result in distortions of spectral shape despite good spatial fidelity. When combined, L1 and SAM provide complementary supervision: SAM constrains the spectral geometry through scale-invariant angular consistency, while L1 anchors the reconstruction to accurate spatial intensity patterns. This complementary gradient behavior explains why their combination yields improved performance across both spectral and spatial metrics.

This theoretical complementarity is reflected in the quantitative results reported in Table 5. The combined L1+SAM loss consistently outperforms the L1-only baseline across all spectral metrics, indicating that integrating angular spectral supervision complements pixel-wise reconstruction and yields more accurate hyperspectral estimates. The parameter α controls the relative importance of the two terms. A larger weight on L1 (α=0.7) achieves the highest PSNR (43.96) and SSIM (98.54), suggesting that stronger pixel-based supervision helps recover sharper spatial details while the SAM term still maintains spectral stability. In contrast, α=0.3 achieves the lowest SAM (0.0249) and $\Delta E_{00}$ (0.063), showing that emphasizing the spectral component improves angular similarity and perceptual color accuracy, with a moderate reduction in spatial metrics.

Training with L1 alone results in the weakest spectral fidelity (SAM = 0.0326, $\Delta E_{00}$ = 0.080), confirming that pixel-wise regression by itself does not fully preserve spectral structure. The TV-based loss mainly acts as a smoothness prior: it reduces noise and improves local consistency, but its gradients are less effective at enforcing precise spectral or spatial correspondence. As a result, its overall accuracy remains between pure L1 and the L1+SAM combinations (SAM = 0.0279, $\Delta E_{00}$ = 0.070). In summary, combining L1 and SAM provides the best overall results. The setting α=0.7 offers the strongest reconstruction in terms of PSNR and SSIM, while α=0.3 is preferable when spectral accuracy and perceptual color fidelity are the primary goals.

#### 5.4.2. Ablation Study on Network Architecture

To motivate the architectural choices of the proposed network, we conduct an ablation study to evaluate the impact of different convolutional blocks and upsampling strategies, analyzing their effect on both reconstruction accuracy and computational efficiency. All experiments are conducted using the best-performing loss function identified in the previous ablation study.

The results visible in Table 6 show that replacing standard convolution with Conv3XC consistently improves reconstruction accuracy without increasing computational cost, as evidenced by the identical GFLOPs when using transposed convolution. This confirms the effectiveness of Conv3XC in enhancing representational capacity while preserving inference efficiency through re-parameterization. A more pronounced performance gain is observed when adopting PixelShuffle instead of transposed convolution, indicating that the choice of upsampling strategy plays a crucial role in high-resolution reconstruction. PixelShuffle leads to substantial improvements in PSNR and SSIM, while requiring additional computational resources. Overall, the combination of Conv3XC and PixelShuffle achieves the best trade-off between reconstruction quality and efficiency, yielding the highest accuracy across all metrics while maintaining a computational cost.

#### 5.4.3. Ablation Study on Robustness to Spatial Misregistration

To evaluate the sensitivity of the proposed method to spatial misalignment between the low-resolution hyperspectral input and the high-resolution RGB guidance, we conduct a controlled ablation study in which we synthetically misalign only the RGB image. The low resolution input and the ground-truth are kept perfectly aligned. This setup mimics realistic acquisition conditions, where independent sensors are calibrated and registered, yet small residual misregistrations inevitably remain [50].

We consider three global geometric transformations applied to the RGB guidance: rotation, translation, and isotropic scaling. For every transformation type, we define a set of distortion levels and, for each image in the test set, we randomly sample the actual transformation parameters from a uniform distribution inside the corresponding range. The transformed RGB is then passed to the network together with the original HSI.

##### Rotation:

We sample an in-plane rotation angle:

θ∼U[−α,α],

with

$$\alpha \in \{0.00^{\circ },\;0.05^{\circ },\;0.10^{\circ },\;0.20^{\circ },\;0.50^{\circ }\}.$$

The affine transformation is applied around the image center, and the resulting image is resampled on the original grid.

##### Translation:

Horizontal and vertical shifts are sampled independently as follows:

Δx,Δy∼U[−T,T],

with

T∈{0.00,0.25,0.50,1.00,2.00}pixels.

##### Scaling:

We apply an isotropic scaling factor:

$$s∼U[s_{\min},s_{\max}],$$

considering the following ranges:

$$\left(s_{\min},s_{\max}\right)\in \left\{\left(1.00,1.00\right),\;\left(0.99,1.01\right),\;\left(0.98,1.02\right)\right\}.$$

Table 7 shows that the method is almost unaffected by rotations up to $0.10^{\circ }$, yielding identical SAM, PSNR and SSIM values to the perfectly aligned case. A noticeable degradation appears for $\alpha =0.20^{\circ }$, and becomes substantial at $0.50^{\circ }$, where PSNR drops by more than 4 dB and SSIM decreases by over 2 points. These results indicate that the network can tolerate sub-degree misregistration, but even moderate rotations introduce spectral and spatial inconsistencies that cannot be fully compensated by the fusion model.

A similar behaviour is observed for translational misalignment as shown in Table 8. Sub-pixel shifts (T≤0.50) have essentially no impact on reconstruction quality, confirming the model’s robustness in the most realistic misalignment regime. However, performance drops sharply for shifts of 1–2 pixels: SAM increases by nearly 15% and PSNR decreases by up to 7 dB compared to the aligned baseline. This highlights that accurate sub-pixel registration between the RGB guidance and the HSI input is crucial for achieving high-quality reconstruction.

The results for isotropic scaling are shown in Table 9. A small scale mismatch of ±1% already leads to a significant drop in PSNR (−6 dB) and SSIM, and a further increase to ±2% results in the worst performance among the three transformations. Unlike rotation and translation, which affect the RGB image locally or directionally, isotropic scaling introduces a global structural discrepancy that systematically misaligns spatial details across modalities. This makes scale mismatch particularly challenging for alignment-dependent fusion architectures.

Overall, these ablation results demonstrate that the proposed approach is robust to very small geometric discrepancies but remains sensitive to larger misregistrations, especially beyond sub-pixel shifts, sub-degree rotations, or scale mismatches exceeding 1%. This emphasizes the importance of accurate cross-sensor calibration and registration when deploying RGB-guided hyperspectral super-resolution in practical settings.

While the proposed robustness analysis provides useful insights into the sensitivity of the model to spatial misregistration, it relies on synthetic global affine perturbations applied to the RGB guidance. This experimental setup approximates realistic multi-sensor acquisition scenarios, where sensors are independently calibrated and residual misalignments typically manifest as small global transformations. However, real-world acquisitions may also exhibit more complex misregistration patterns, including locally varying distortions caused by optical differences, rolling shutter effects, lens distortions, or scene-dependent parallax, which cannot be fully captured by global affine models. Non-rigid or spatially varying misalignments, such as local warping or depth-induced parallax, are not explicitly addressed in this study. Handling such cases would likely require either explicit deformable alignment mechanisms or architectures that are less dependent on strict pixel-wise correspondence between modalities. An interesting direction for future work is the integration of learned alignment strategies, such as deformable convolution or feature-level cross-modal alignment modules, which could enable the network to compensate for more complex misregistration patterns. Alternatively, joint optimization of alignment and super-resolution within a unified framework may further improve robustness in unconstrained acquisition settings.

## 6. Conclusions

In this work, we presented CGNet, a multiscale RGB-guided fusion framework for hyperspectral image super-resolution, designed to exploit the spatial richness of RGB imagery while preserving the spectral fidelity of the hyperspectral signal.

Extensive experimental results demonstrate that CGNet consistently outperforms state-of-the-art approaches on multiple benchmarks, achieving superior performance in terms of SAM, PSNR, SSIM, and $\Delta E_{00}$. In particular, the proposed method exhibits strong spectral preservation capabilities, confirming its effectiveness in maintaining spectral consistency while enhancing spatial details.

While PSNR, SSIM, SAM, and $\Delta E_{00}$ are widely adopted for benchmarking hyperspectral super-resolution methods and provide a necessary first-order assessment of reconstruction fidelity, we acknowledge that such generic metrics do not fully capture task-specific utility in downstream applications. In practice, small spectral or spatial reconstruction errors may have disproportionate effects on subsequent tasks such as material classification, spectral unmixing, or medical image analysis, where subtle spectral variations can be critical. Investigating the relationship between reconstruction quality and downstream task performance therefore represents an important direction for future research.

We further conducted an extensive experimental analysis to better understand the practical behavior of CGNet beyond reconstruction accuracy. In particular, the computational efficiency study highlights that, despite a higher nominal model complexity in terms of parameters and GFLOPs compared to some lightweight designs, CGNet achieves one of the fastest inference times among learning-based RGB-guided approaches. This demonstrates that real-world efficiency is strongly influenced by architectural design and data-flow organization rather than theoretical complexity alone, and confirms the suitability of CGNet for practical deployment.

In addition, a comprehensive set of ablation studies was performed to analyze the contribution of the loss formulation, architectural components, and robustness to spatial misregistration. The loss ablation confirms the complementary roles of L1 and SAM supervision in jointly preserving spatial structures and spectral shape. The architectural ablation validates the effectiveness of the proposed Conv3XC blocks and PixelShuffle-based upsampling in achieving a favorable balance between reconstruction accuracy and efficiency. Finally, the misregistration ablation demonstrates that CGNet remains stable under very small geometric deviations, while performance degrades when misalignment exceeds sub-degree rotations, sub-pixel translations, or scale discrepancies above 1% Together, these analyses provide a clearer understanding of the design choices and practical limitations of the proposed framework.

Overall, CGNet provides an effective and computationally efficient solution for RGB-guided hyperspectral image super-resolution. Future work will focus on improving robustness to complex cross-modal misalignments by integrating explicit alignment modules and self-supervised registration strategies. In particular, incorporating learnable geometric transformation components, such as homography estimation, deformable convolutions, or optical-flow-based warping networks, may further enhance spatial consistency under realistic acquisition conditions.

## Figures and Tables

**Table 1:** Comparison of methods at ×4 and ×6 scales for ARAD1K. Metrics include SAM, PSNR, SSIM, and $\Delta E_{00}$. For each metric, for each scale factor, best results in bold. The arrows indicate the optimization direction: ↑ means higher is better, while ↓ means lower is better.

Scale	Color Guidance	Model	SAM↓	PSNR↑	SSIM↑	$\Delta E_{00}$↓
×4	No	Bicubic	0.031	33.21	88.60	0.165
		RCAN [6]	0.039	36.61	93.12	0.148
		SSPSR [8]	0.027	36.56	92.91	0.151
		EDSR [7]	0.029	37.25	93.70	0.143
	Yes	u2MDN [10]	0.032	42.76	97.99	0.092
		NonReg [11]	0.026	43.04	98.02	0.090
		Integrated [12]	0.027	43.60	98.29	0.083
		Ours	**0.025**	**43.72**	**98.33**	**0.071**
×6	No	Bicubic	0.040	30.99	83.32	0.210
		RCAN [6]	0.050	33.51	88.25	0.185
		SSPSR [8]	0.037	33.59	88.42	0.182
		EDSR [7]	0.040	34.02	89.10	0.176
	Yes	u2MDN [10]	0.043	39.04	95.79	0.127
		NonReg [11]	0.035	39.29	96.02	0.119
		Integrated [12]	0.037	39.79	96.36	0.115
		Ours	**0.031**	**41.96**	**97.72**	**0.102**

**Table 2:** Comparison of methods at ×4 and ×6 scales for StereoMSI. Metrics include SAM, PSNR, SSIM, and $\Delta E_{00}$. For each metric, for each scale factor, best results in bold. The arrows indicate the optimization direction: ↑ means higher is better, while ↓ means lower is better.

Scale	Color Guidance	Model	SAM↓	PSNR↑	SSIM↑	$\Delta E_{00}$↓
×4	No	Bicubic	0.131	35.28	93.04	0.215
		RCAN [6]	0.199	33.28	92.21	0.242
		SSPSR [8]	0.197	35.79	92.91	0.230
		EDSR [7]	0.189	37.25	93.58	0.221
	Yes	u2MDN [10]	0.110	37.16	94.21	0.180
		NonReg [11]	0.146	37.63	94.23	0.175
		Integrated [12]	0.103	38.15	94.97	0.162
		Ours	**0.085**	**39.24**	**96.60**	**0.140**
×6	No	Bicubic	0.185	31.12	84.10	0.280
		RCAN [6]	0.127	31.45	84.75	0.265
		SSPSR [8]	0.160	31.90	84.73	0.258
		EDSR [7]	0.120	31.76	85.46	0.250
	Yes	u2MDN [10]	0.115	33.51	89.65	0.215
		NonReg [11]	0.151	33.59	89.12	0.210
		Integrated [12]	0.118	34.02	90.73	0.195
		Ours	**0.090**	**38.55**	**96.25**	**0.160**

**Table 3:** Cross-dataset evaluation on StereoMSI when training on ARAD1K. All compared methods leverage RGB guidance. Metrics include SAM, PSNR, SSIM and $\Delta E_{00}$. Best results for each scale are highlighted in bold. The arrows indicate the optimization direction: ↑ means higher is better, while ↓ means lower is better.

Scale	Model	SAM↓	PSNR↑	SSIM↑	$\Delta E_{00}$↓
×4	u2MDN	0.275	29.16	86.21	0.378
	NonReg	0.365	29.63	86.23	0.368
	Integrated	0.258	30.15	86.97	0.340
	Ours	**0.213**	**31.24**	**89.60**	**0.294**
×6	u2MDN	0.288	25.51	79.65	0.452
	NonReg	0.378	25.59	79.12	0.441
	Integrated	0.295	26.02	80.73	0.410
	Ours	**0.225**	**30.55**	**87.25**	**0.336**

**Table 4:** Computational efficiency and reconstruction performance comparison at ×4 scale on the ARAD1K dataset. Reported metrics include spectral accuracy (SAM), spatial fidelity (PSNR, SSIM), model complexity (number of parameters and GFLOPs), and inference time per image. Best results for each metric are highlighted in bold, second best results underlined. The arrows indicate the optimization direction: ↑ means higher is better, while ↓ means lower is better.

Scale	Color Guidance	Model	SAM↓	PSNR↑	SSIM↑	Params (M)↓	GFLOPs↓	Time (S)↓
×4	No	Bicubic	0.031	33.21	88.60	/	/	0.001
		RCAN [6]	0.039	36.61	93.12	15.62	245.2	0.127
		SSPSR [8]	0.027	36.56	92.91	22.52	902.6	0.370
		EDSR [7]	0.029	37.25	93.70	61.51	776.7	0.640
	Yes	u2MDN [10]	0.032	42.76	97.99	**0.01**	**10.48**	0.710
		NonReg [11]	0.026	43.04	98.02	2.13	96.6	**0.019**
		Integrated [12]	0.027	43.60	98.29	/	/	238.0
		Ours	**0.025**	**43.72**	**98.33**	3.16	113.10	0.034

**Table 5:** Ablation study on different loss functions. Metrics include SAM, PSNR, SSIM, and $\Delta E_{00}$. Best results for each metric are highlighted in bold. The arrows indicate the optimization direction: ↑ means higher is better, while ↓ means lower is better.

Loss	SAM↓	PSNR↑	SSIM↑	$\Delta E_{00}$↓
$L_{L1+\mathrm{SAM}}^{\left(\alpha =0.3\right)}$	**0.0249**	42.75	97.98	**0.063**
$L_{L1+\mathrm{SAM}}^{\left(\alpha =0.5\right)}$	0.0250	43.65	98.36	0.067
$L_{L1+\mathrm{SAM}}^{\left(\alpha =0.7\right)}$	0.0255	**43.96**	**98.54**	0.069
$L_{L1}$	0.0326	43.40	98.26	0.080
$L_{\mathrm{TV}}$	0.0279	43.11	98.06	0.070

**Table 6:** Ablation study on architectural design choices. We compare standard convolution and Conv3XC blocks combined with different upsampling strategies. Metrics include SAM, PSNR, SSIM, and GFLOPs. Best results for each metric are highlighted in bold. The arrows indicate the optimization direction: ↑ means higher is better, while ↓ means lower is better.

Block	Upsampling	SAM↓	PSNR↑	SSIM↑	GFLOPs↓
Conv2D	ConvTranspose	0.0351	40.25	95.18	**100.74**
Conv3XC	ConvTranspose	0.0308	41.99	96.10	**100.74**
Conv2D	PixelShuffle	0.0280	42.70	97.70	113.10
Conv3XC	PixelShuffle	**0.0255**	**43.96**	**98.54**	113.10

**Table 7:** Ablation study on rotational misregistration of the RGB guidance. Metrics include SAM, PSNR, and SSIM. The arrows indicate the optimization direction: ↑ means higher is better, while ↓ means lower is better.

α	SAM↓	PSNR↑	SSIM↑
$0.00^{\circ }$	0.0250	43.72	98.33
$0.05^{\circ }$	0.0250	43.72	98.33
$0.10^{\circ }$	0.0250	43.72	98.33
$0.20^{\circ }$	0.0256	42.27	97.83
$0.50^{\circ }$	0.0261	39.56	96.32

**Table 8:** Ablation study on translational misregistration of the RGB guidance. Metrics include SAM, PSNR, and SSIM. The arrows indicate the optimization direction: ↑ means higher is better, while ↓ means lower is better.

T	SAM↓	PSNR↑	SSIM↑
0.00	0.0250	43.72	98.33
0.25	0.0250	43.72	98.33
0.50	0.0250	43.72	98.33
1.00	0.0271	39.02	96.01
2.00	0.0283	36.09	92.26

**Table 9:** Ablation study on isotropic scaling misregistration of the RGB guidance. Metrics include SAM, PSNR and SSIM. The arrows indicate the optimization direction: ↑ means higher is better, while ↓ means lower is better.

s	SAM↓	PSNR↑	SSIM↑
(1.0, 1.0)	0.0250	43.72	98.33
(0.99, 1.01)	0.0270	37.67	95.11
(0.98, 1.02)	0.0291	36.02	91.40

## Data Availability

The data presented in this study are openly available in GitHub at https://github.com/boazarad/ARAD_1K (accessed 24 January 2026) and in CSIRO Data Access Portal at https://data.csiro.au/collection/csiro:40743 (accessed 24 January 2026).
